# Continuous non-invasive electrophysiological monitoring in high-risk pregnancies: study protocol of a cohort intervention random sampling study in a tertiary obstetrical care centre in the Netherlands (NIEM-O study)

**DOI:** 10.1136/bmjopen-2025-102732

**Published:** 2025-11-12

**Authors:** Nadine D de Klerk, Phebe B Q Berben, Ivar R de Vries, Hendrik Niemarkt, Rik Vullings, Edwin R van den Heuvel, Myrthe van der Ven, Annemarie F Fransen, S Guid Oei, Judith O E H van Laar

**Affiliations:** 1 Gynaecology and Obstetrics, Maxima Medical Centre, Veldhoven, Netherlands; 2 Electrical Engineering, Eindhoven University of Technology, Eindhoven, Netherlands; 3 Pediatrics, Maxima Medical Centre, Veldhoven, Netherlands; 4 Mathematics & Computer Science, Technische Universiteit Eindhoven, Eindhoven, Netherlands; 5 Faculty of Biomedical Engineering, Eindhoven University of Technology, Eindhoven, Netherlands

**Keywords:** OBSTETRICS, Pregnant Women, PERINATOLOGY, Fetal medicine, Pregnancy

## Abstract

**Introduction:**

Women with high-risk pregnancies (eg, pre-eclampsia, imminent preterm birth) are often hospitalised due to the need for foetal and maternal monitoring. They are monitored for 30–45 min up to three times a day with conventional cardiotocography (CTG). In the meantime, they reside at the hospital, but the foetal status is not monitored. Continuous foetal monitoring is currently not recommended using CTG, due to the potential temperature rise from consistent exposure to ultrasound waves. For safe 24/7 monitoring, newly developed devices using non-invasive electrophysiological cardiotocography (eCTG) instead of conventional CTG offer a promising alternative. Previous research into eCTG has shown favourable results in monitoring foetal heart rate throughout both pregnancy and labour. This study aims to investigate the effect of implementing continuous antepartum eCTG monitoring in hospitalised high-risk pregnancies on perinatal and maternal outcome.

**Methods and analysis:**

In this single centre prospective cohort intervention random sampling study, eligible women will be included on the Obstetric High Care of Máxima MC Veldhoven, the Netherlands. In total, 511 pregnant women with a singleton pregnancy between 23+0 and 32+0 weeks of gestation requiring hospitalisation will be recruited. Eligible women will be prospectively included in the cohort receiving standard treatment: intermittent CTG monitoring. From these women, a random sample of the prospective cohort will be offered a new monitoring method: 24/7 eCTG monitoring. For the eCTG monitoring, a wireless abdominal electrode patch, the Nemo Foetal Monitoring System will be used. Additional data from a historical cohort (2014–2019) of 1400 women receiving standard treatment will be included. Perinatal and maternal outcome, along with satisfaction levels of both patient and caregivers, will be compared between groups.

**Ethics and dissemination:**

The study is registered on 18 October 2022 to the Central Committee on Research Involving Human Subjects (NL82869.015.22) via https://www.toetsingonline.nl/to/ccmo_monitor.nsf/conceptabr?OpenForm and approved by the Medical Ethics Committee of Máxima MC (W22.070) on 7 November 2023. Results of the study will be disseminated in peer-reviewed scientific journals and conference presentations.

**Trial registration number:**

NCT06151613.

**STRENGTHS AND LIMITATIONS OF THIS STUDY**

- Continuous antepartum cardiotocographic (CTG) monitoring is implemented using a CE (Conformité Européenne)-certified device already in clinical use, and is compared to standard care (intermittent CTG monitoring).
- The cohort intervention random sampling study design allows inclusion of both prospective and retrospective data, reducing study exposure.
- Signal quality of electrophysiological CTG (eCTG) may be reduced between 28 and 32 weeks of gestation due to vernix caseosa, potentially requiring a switch to conventional CTG.
- Signal loss and switch rate between monitoring methods are systematically documented.
- Interpretation of preterm eCTG traces is based on literature-informed standard operating procedure and daily expert review.

## Introduction

 During pregnancy, maternal or foetal complications might arise, putting the mother and fetus in danger. When complications are diagnosed, pregnant women are often hospitalised for close monitoring of the maternal and foetal condition. One of the tools widely used to determine foetal well-being is cardiotocography (CTG). This is a combined external measurement method using two transducers that measure foetal heart rate (FHR) by the use of Doppler ultrasound (DU) and uterine activity by the use of tocodynamometry (TOCO). Despite its global utilisation, CTG has certain limitations. One of the drawbacks is the poor signal quality of DU and TOCO due to maternal and foetal movements. This arises particularly when monitoring preterm fetuses, multiple gestations and women with high body mass index (BMI).^1^,^4^ Additionally, despite the widespread use of CTG, there has been no reduction in the perinatal mortality rate. The above-mentioned limitations of CTG raise many questions as to how foetal monitoring can be optimised to improve perinatal outcomes. Due to a knowledge gap regarding preterm antepartum monitoring, clinical practice guidelines for CTG monitoring of preterm fetuses have not yet been developed. However, there are observational studies about antepartum CTG monitoring. These observational studies suggest that non-reassuring CTG traces are associated with adverse perinatal outcomes.^5^ The time interval between non-reassuring antepartum CTG traces and sudden foetal death can be only days to hours.^6^,^8^ This short time interval makes the current intermittent use of conventional CTG monitoring (one to three times a day for 30–45 min) inadequate for timely detection of foetal compromise. The use of continuous monitoring could potentially be a solution for optimising perinatal outcomes in preterm antepartum care. Not much is known about continuous antepartum monitoring. Nonetheless, a recent study claims twenty-four-hour CTG was found to be a good screening method to detect early onset hypoxia and thus recognise a fetus at distress early.^9^ Conventional CTG monitoring could not be used continuously in this study, due to the possibility of rise in foetal tissue temperature, resulting from constant exposure to ultrasound waves.^10 11^ Therefore, a new monitoring technique was used. Using this new technique based on electrophysiology, it was possible to safely assess FHR continuously.^12^ One of the devices that enables continuous non-invasive electrophysiological CTG (eCTG) monitoring is the NFMS (Nemo Foetal Monitoring System).^13 14^ In addition to the potential for continuous monitoring by eCTG, it has several additional advantages over conventional CTG monitoring. First, the intrapartum use of eCTG showed a reliable FHR in >95% of the time.^12^ When comparing this to DU used in conventional CTG monitoring, eCTG is far more reliable and accurate.^12^ Whether the eCTG also provides a reliable and accurate registration for preterm antepartum surveillance requires further examination. Second, eCTG monitoring is especially suited for the increasing number of women with obesity, where conventional CTG monitoring often fails.^23 15^,^18^ Additionally, there is no need to reposition the patch, whereas the transducers of conventional CTG often need to be repositioned due to the movements of the fetus. Disadvantages of eCTG are that preparation of the abdomen with abrasive paper can cause skin irritation and that eCTG cannot be used by women with a pacemaker, due to possible signal interference. Moreover, the performance of eCTG might be reduced in women between 28 and 32 weeks of gestation, due to lower quality of the non-invasive foetal electrocardiographic (NI-fECG) recordings resulting from the vernix caseosa impeding signal transmission.^19^ These (dis)advantages must be taken into account when introducing eCTG in clinical care. In order to improve perinatal outcomes through prevention of unnecessary or delayed interventions by implementing continuous monitoring, we set up the NIEM-O study (non-invasive electrophysiological monitoring on the Obstetric High Care). The aim of the study is to evaluate the effect of continuous antepartum eCTG monitoring on severe perinatal morbidity and mortality, by comparing with conventional intermittent CTG monitoring.

## Methods and analysis

The study is a single centre, prospective cohort intervention random sampling study (CIRSS) with historical controls: the NIEM-O study. Pregnant women will be recruited from the Máxima Medical Centre (MMC), a tertiary obstetric care hospital in Veldhoven, the Netherlands. Inclusion starts at the end of 2023, and is expected to take at least 2 years. Pregnant women will be recruited between 23 and 32 gestational age (GA) when they are admitted to the obstetric high care (OHC). The study protocol (version 9) is approved by the Medical Ethics Committee of Máxima MC (W22.070/NL82869.015.22).

### Study objectives

The primary objective of the study is to evaluate the effect of continuous antepartum eCTG monitoring on severe perinatal morbidity and mortality, by comparing with conventional intermittent CTG monitoring. The objectives are investigated in preterm fetuses (GA 23–32 weeks) whose mothers are admitted to the OHC. The primary study outcome is a composite outcome consisting of (1) Perinatal mortality, defined by the WHO as: deaths that occur after a gestational age of ≥22 weeks or, if unknown >500 gram, until 28 days after birth^20^ and (2) Major neonatal morbidity assessed at the time point when the neonate is discharged from the hospital.^21^ Major neonatal morbidity is defined as either: intraventricular haemorrhage (IVH) grade three or more,^22 23^ periventricular leukomalacia (PVL) grade two or more,^24 25^ moderate or severe bronchopulmonary dysplasia (BPD),^26^,^28^ necrotising enterocolitis (NEC) grade two or more,^29 30^ or retinopathy of prematurity (ROP) necessitating laser therapy.^31^ Secondary study objectives are the effect of continuous antepartum eCTG compared with conventional intermittent CTG monitoring on maternal mortality, neonatal morbidity, satisfaction for both patient and caregiver, duration of pregnancy, the need to switch between monitoring methods (either from eCTG to CTG or vice versa), timing and number of obstetric interventions, duration of admission at the OHC and admission and duration of admission at the neonatal intensive care unit. Demographics will be obtained from the electronic patient file. The secondary outcomes are defined as follows: maternal mortality as defined by the WHO^32^: any death from any cause related to or aggravated by pregnancy or its management (excluding accidental or incidental causes) during pregnancy and childbirth or within 42 days of termination of pregnancy, irrespective of the duration and the site of the pregnancy. Neonatal morbidity, excluding major neonatal morbidity described as primary outcome, is defined as: Any health condition attributed to and/or aggravated by birth, assessed at the time point when the neonate is discharged from the hospital^21^ or—if discharged within 4 weeks after birth—at 4 weeks after birth, that has negative outcomes to the neonatal well-being (ie, IVH grade 1–2, PVL grade 1, mild BPD, NEC grade 1, ROP (any grade)) not necessitating laser therapy, hypoxic ischaemic encephalopathy, neonatal seizures, neonatal sepsis (culture proven), need for intubation on the delivery room, mechanical ventilation within the first 72 hours after birth, antibiotics within the first 72 hours after birth, spontaneous intestinal perforation necessitating surgery, surfactant treatment, Apgar score <7 after 5 min, severe metabolic acidosis (pH <7.05 and base deficit ≥12 mmol/L). This definition of severe metabolic acidosis is set at pH <7.10 and base excess ≥12 mmol/L in cases with only an umbilical vein sample (one available blood gas sample or the pH difference between two samples below 0.03^33^). Next to the study objectives described above, the data that is gathered in this study will be used to develop and verify mathematical models that aim at the prediction of time to (premature) birth and at automated detection of foetal movements.

### Population

To be eligible to participate in this study, women must be 18 years or older, with a singleton pregnancy between 23+0 and 32+0 weeks of gestation that requires hospitalisation to the OHC for foetal monitoring and parents wishing for foetal monitoring (table 1). Exclusion criteria are multiple pregnancy, contraindications to abdominal patch placement (eg, abdominal skin disease), women connected to external or implanted electrical stimulators, foetal or maternal cardiac arrhythmias, insufficient knowledge of Dutch or English language, women admitted with a clinical diagnosis of sepsis with hypotension (ie, septic shock), or when the treatment plan (with intervention planned within 24 hours) is already made before inclusion has been completed (table 1). Included participants can withdraw from study participation at any time without giving reason and without any consequences.

**Table 1:** Inclusion and exclusion criteria

Inclusion criteria	Exclusion criteria
Maternal age ≥18 years	External or implanted electrical stimulator
Singleton pregnancy	Multiple pregnancy
GA 23+0–32+0 weeks	Foetal or maternal cardiac disease
Requiring hospitalisation to the OHC for foetal surveillance	Contraindication to abdominal patch placement (eg, abdominal skin disease)
Parents wishing for foetal monitoring	Intervention planned <24 hours before inclusion is completed
	Women admitted with a clinical diagnosis of sepsis with hypotension (ie, septic shock).
	Insufficient knowledge of Dutch or English language

GA, gestational age; OHC, obstetric high care.

### Patient and public involvement

Care4Neo, Neonatal Patient and Parent Advocacy Organization, Rotterdam, The Netherlands, reviewed the study protocol from a patient and parent perspective in the funding stage. They also reviewed and contributed to the patient information leaflet.

### Study design

**Figure 1:**
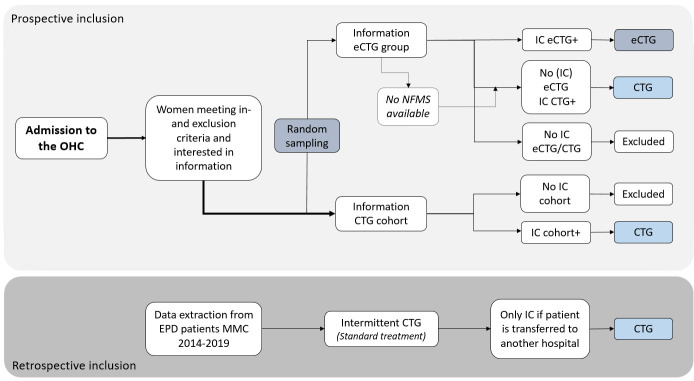
Cohort intervention random sampling study (CIRSS) design. Informed consent (IC) is asked after the patient is sampled or not. CTG, cardiotocography; eCTG, electrophysiological cardiotocography; MMC, Máxima Medical Centre; NFMS, Nemo Foetal Monitoring System; OHC, obstetric high care, EPD, electronic patient documentation.

The CIRSS was chosen to balance methodological rigour with feasibility and ethical considerations in a high-risk obstetric population. This design feature has recently been described and compared with other study designs^34^, highlighting its ability to combine real-world applicability with robust statistical correction for potential biases. The use of historical controls and random sampling within a prospective cohort allows for efficient implementation and broader inclusion, while maintaining internal validity through causal inference techniques such as inverse probability weighting with a per-protocol analysis. This CIRSS design has two components: a prospective data collection and a retrospective data collection (figure 1).

#### Prospective recruitment

**Figure 2:**
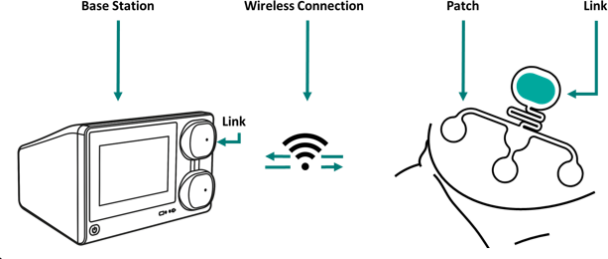
The Nemo Foetal Monitoring System (NFMS) consists of three components: base, link and patch (source: user manual NFMS).

Potential participants will be informed about the study by their obstetric healthcare professional when admitted to the hospital. First, the healthcare professionals will verify the inclusion and exclusion criteria (table 1), after which the women are added to the study using an encryption key. These women are part of the cohort study and receive standard care consisting of conventional intermittent CTG monitoring. A part of these women will be randomly sampled. This random sampling is conducted by the research team using a secure algorithm embedded in Research Manager (ISO 27001 certified). They will be offered the new treatment: continuous eCTG monitoring using the NFMS (figure 2). The women will either be part of the cohort study, thus receiving standard care consisting of conventional intermittent CTG monitoring, or they will be sampled for continuous eCTG monitoring using the NFMS. Appropriate explanation and written study information will be provided hereafter by the healthcare professional, and they will ask the researchers for further counselling. Non-sampled women will only receive information regarding the collection of their data in the cohort study. Sampled women will be informed about receiving continuous eCTG monitoring. A researcher will complete the informed consent (IC) procedure, including collecting written consent (for an example of the participant consent form, see online supplemental file 1). When the women do not wish to participate in the study, they will be excluded, their encrypted information will be obliterated and the exclusion will be anonymously documented. Once women are included, they are given the appropriate monitoring method. Five NFMS devices are available at the OHC of Máxima MC. When no NFMS device is available within 24 hours after sampling and inclusion, the sampled women will receive standard treatment within the study. For continuous monitoring, trained nursing staff will oversee device placement and signal quality checks during routine care. CTG traces are continuously displayed on a central monitoring system, where deviations from normal patterns trigger automated alarms to alert clinical staff. In addition, CTG traces are systematically assessed by physicians at least four times per 24 hours at predefined intervals, partly retrospectively, and more frequently if clinically indicated. These assessments follow a standard operating procedure (SOP) to ensure consistency and clinical relevance. If the eCTG recording does not provide sufficient signal quality for reliable monitoring, we will report this and, if needed, can be switched to standard CTG monitoring. This decision will be made on the clinical judgement of the attending gynaecologist. No literature was available on an acceptable signal loss threshold. The International Federation of Gynaecology and Obstetrics Guidelines advises switching monitoring methods in intrapartum monitoring when a signal loss threshold of 20% is reached.^35^ Based on this, a standardised protocol for switching to conventional CTG will be implemented when signal loss exceeds 20%, or when clinical judgement deems the eCTG trace insufficient for reliable interpretation. This protocol will be documented and monitored throughout the study. Women who decline to be part of the cohort will receive standard care: conventional CTG monitoring without data collection for research. All prospectively included women will receive a digital invitation to two digital surveys. Women are asked to fill in the first survey about their experience with the received monitoring method—EQ5D5L questionnaire^36^—on the first day after admission, and the second survey about their experience—D-QUEST^37^—on the last day of admission. Although the patient satisfaction surveys are administered at admission and discharge, all participants are followed up until (at least) 4 weeks postpartum. During this period, patients have the opportunity to share additional reflections or concerns with their care team, which may provide further qualitative insights into their experience with the monitoring method.

Additionally, for the development and validation of a mathematical model regarding the automated detection of foetal movement, the first 200 consecutive women monitored with NFMS will receive a parallel 45 min ultrasound recording extended with a six-channel accelerometric measurement. The ultrasound will be annotated in real-time for foetal movement and will be performed within 4 days following admittance.

Finally, if patients are admitted to the OHC repeatedly, they stay in the same monitoring group, not allowing cross-over with the other group. Caregivers will receive a validated digital survey designed by Brown *et al*^38^ at the start of the study and after 1 year to evaluate their satisfaction regarding the monitoring methods.

#### Retrospective recruitment

In order to strengthen the comparison of the two groups (continuous eCTG monitoring and conventional intermittent CTG monitoring), additional data from women who received standard treatment (conventional intermittent CTG monitoring) in Máxima MC Veldhoven, the Netherlands in 2014–2019 will be collected retrospectively. Data will be derived from electronic patient records (EPRs).

#### Data collection

All required demographic and medical information will be obtained from the EPR at the Máxima MC or the referring hospital, when the patient has been transferred to or from this hospital before or after their admission to the OHC. This includes maternal and perinatal information. For the mother: age, ethnicity, BMI, obstetric history, general medical history, medication use, intoxications, information about the course of pregnancy, complications during pregnancy, delivery and (until 6 weeks) postpartum, For the fetus/neonate: ultrasound results such as foetal biometry, structural abnormalities or Doppler measurements, gestational age at delivery, gender, birth weight, admission to neonatal ward and the reason for admission, Apgar scores, pH and base excess in umbilical artery and vein, mortality, morbidity, general information on neonatal health in the first 4 weeks postpartum. Research Manager (ISO 27001 certified) is used to store patient data.

### Intervention

Sampled women will receive continuous eCTG monitoring (when accepted), using the NFMS. The system is CE (Conformité Européenne) -licensed and already in use as standard care in several hospitals throughout Europe. The NFMS consists of a base, link and abdominal patch (figure 2). Corresponding to the manufacturer’s instructions, the electrode patch will be applied by trained personnel and employed inside the uterus boundaries. Before application of the patch, the abdominal skin must be washed thoroughly with water and soap and subsequently gently abraded with medical abrasive paper. This procedure is necessary to achieve low skin impedance and ensure good electrical conduction of the foetal heart signal.^39^ Every recording will continue 24/7, with a minimum of 21 hours per 24 hours.

### Sample size

**Figure 3:**
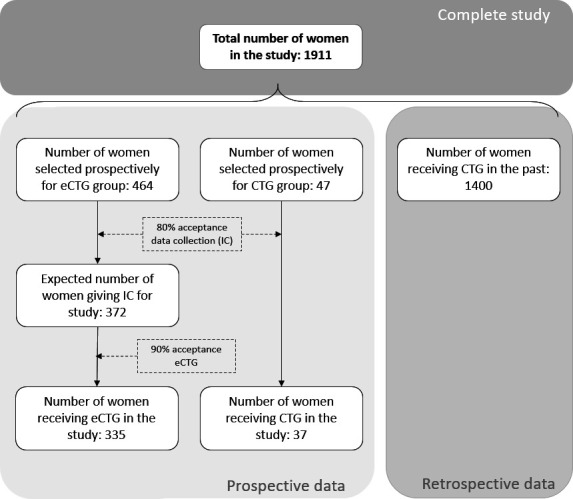
Flowchart of sample size calculation including estimation of informed consent (IC) and eCTG acceptance rates. CTG, cardiotocography; eCTG, electrophysiological cardiotocography.

A total of 1911 patients (comprised of 511 prospectively included women and 1400 historical controls) would be sufficient to evaluate the primary objective. Incidence of the composite outcome is based on numbers of perinatal morbidity and mortality of MMC from 2019, combined with a literature-based estimation of the simultaneous occurrence of the perinatal comorbidities and mortality.^40^,^43^ Due to the unknown effect of COVID on perinatal and maternal outcomes,^44^ numbers from MMC of 2020–2021 are not included in the sample size calculation. This resulted in a rounded estimated composite outcome of 17%. Based on clinical experience, an effect size of 6% is expected. To detect this effect size with a power of 80% and a type I error of 5%, a group of 511 women will be invited for the prospective cohort. Of these 511 women, 464 women (90.8%) will be in the sampled study arm and 47 women (9.2%) in the non-sampled study arm. Based on clinical experience, an estimation of 80% acceptance rate for data collection in the sampled and non-sampled group was made (resulting in 372 women and 37 women respectively). Among the 372 women who agreed to data collection in the sampled group, it is expected that 90% will accept and receive eCTG monitoring (335 women) and 10% (37 women) will decline eCTG monitoring and therefore receive CTG monitoring. All 37 women in the non-sampled group will receive standard monitoring. For the retrospective cohort, a total of 1400 participants will be included (figure 3). No dropout rate is calculated, due to continuation of the study and sampling until the requested sample size (of both groups) is reached.

### Statistical analysis

For the primary and secondary outcomes, based on the demographics, characteristics of the women and their baseline outcomes, a propensity score will be created. This will be used to correct for possible selection bias that may occur due to (1) the group of women who are offered eCTG but receive CTG (either by choice or circumstances) or (2) the retrospective and prospective nature of the study. Thus, a per-protocol analysis with propensity score adjustment will be considered. The propensity score models will include maternal characteristics (eg, age, BMI, parity, ethnicity), foetal indicators (eg, gestational age, foetal growth restriction, Doppler abnormalities) and care-related variables (eg, indication for admission, medication use, duration of hospitalisation). These covariates were selected based on clinical relevance and availability across both cohorts. For categorical outcomes, a logistic regression analysis will be applied to estimate the treatment effect corrected for the propensity score. For numerical outcome values, a general linear model will be applied with an appropriate probability distribution link function and the sandwich estimator to estimate a treatment effect corrected for the propensity score. For time-to-event outcome, a Cox proportional hazards model will be used with a sandwich estimator to estimate a treatment effect corrected for the propensity score. For the data gathered from the questionnaires, standard statistical tests will be used (χ^2^, t-test, Mann-Whitney tests) to determine a difference between the two treatment groups. A propensity score approach will be applied as well if large imbalances between characteristics are present between CTG and eCTG participants. Several sensitivity analyses are being anticipated. The combined endpoint will be analysed as separate end-points. Several subgroup analyses are investigated (eg, obesity). A p value of <0.05 is considered statistically significant unless otherwise specified. When possible, 95% CIs will be calculated for the reported measures of effect. Missing data will be evaluated and reported. Limited missing data is expected, and if missingness is less than 5%, list-wise deletion will be used. Otherwise, multiple imputation (eg, predictive mean matching) will be used. The imputation will be repeated at least 10 times and Rubin’s rule will be used to combine estimates and standard errors from the imputed data.

## Ethics and dissemination

### Ethics approval and consent to participate

This study protocol and execution of the study will be fully compliant with the most recently updated version of the Declaration of Helsinki. The researchers will protect confidential data in accordance with the rules and regulations stated in the Dutch General Data Protection Regulation. The method for data storage and handling is approved by the data security officer of Máxima MC. The Medical Ethics Committee of Máxima MC extensively reviewed the study protocol and concluded that the study is in line with the Medical Research Involving Human Subjects Act. Therefore, ethical approval was granted (W22.070/NL82869.015.22). Máxima MC Board of Management also approved the conduct of this study at their hospital. Written informed consent will be obtained from all subjects involved in the prospective part of the study and from all the participants who were transferred to/from another hospital and who are part of the retrospective part of the study. The Clinical Trial Centre Maastricht, acting as an independent monitor, has categorised this study as having ‘Negligible’ risk, resulting in a minimum of seven monitoring visits throughout the study.

### Ethics and risk assessment

Only a relatively small possibility of moderate skin irritation or of a minor allergic reaction resulting from abrading the abdominal skin or applying conductive gel can be anticipated for correct application of the NFMS patch. However, there is limited experience with long-term application of the patch. Skin irritation will be reported as an adverse outcome and will be monitored closely during the study. If necessary, a new patch can be placed on a different site on the abdomen or a switch to conventional CTG monitoring can be made. These women will still be part of the study.

### Dissemination

Results of the study will be disseminated in peer-reviewed scientific journals and conference presentations.

## Discussion

The NIEM-O study investigates the effect of continuous antepartum eCTG monitoring compared with conventional intermittent CTG monitoring, in preterm hospitalised high-risk pregnancies, on perinatal and maternal outcome. Our hypothesis is that the novel monitoring technique (eCTG) and its continuous antepartum use may lead to earlier detection of foetal distress and may prevent unnecessary interventions. This might result in improved perinatal and maternal outcomes. It also provides more insight into preterm and continuous antepartum foetal monitoring. This will support consensus and recommendations regarding the interpretation of antepartum foetal monitoring for preterm fetuses.^45 46^ Similarly, limited knowledge exists regarding the utilisation and interpretation of continuous (electrophysiological) foetal monitoring (eCFM) for a prolonged period of time.^47^ The comprehensive nature of this study’s database renders it suitable for future research inquiries and as a reference database.

A similar large-scale clinical study (NIEM-II study) is currently being conducted in the term population, comparing the effect of eCTG with conventional CTG monitoring during labour on various perinatal and maternal outcomes.^48^ Both studies—this NIEM-O study and the NIEM-II study—employ the same CIRSS design, enabling efficient implementation and comparison of eCTG with standard monitoring in routine clinical care across different obstetric populations.

### Study limitations

It is unknown if reference standards for CTG patterns of term fetuses as stated in current literature can be applied to fetuses during the preterm period.^46^ Also, little to no research is available on the use and interpretation of eCFM for a prolonged period of time.^47^ Before study initiation, all clinical staff involved in CTG interpretation will undergo standardised training sessions based on current literature and internal guidelines.^4649^,^53^ Additionally, daily multidisciplinary meetings will be held to discuss CTG traces, fostering consensus and calibration among attending gynaecologists. These procedures will be documented in an SOP. Accurate determination of the foetal condition based on the CTG trace may be subject to inter-observer variability, possibly influencing perinatal outcome.^46^ However, this concerns both the use of conventional intermittent CTG as well as the continuous eCTG monitoring method. The vernix caseosa, a fatty layer covering the fetus between 28 and 32 weeks GA, may dim the foetal cardiac signal and might lead to insufficient data for NI-fECG analysis.^39^ This possible decrease in signal quality might result in a necessity to switch to conventional CTG monitoring. No risks are involved in switching to the conventional system. Signal loss and switch rate will be documented and are part of the secondary objectives of the study. The CIRSS study design deviates from a standard randomised clinical trial, where both treatment arms are randomised to patients. The inclusion rate of patients is expected to be substantially larger than for a randomised controlled trial, since (almost) all women who participate and receive standard care are automatically included. By making use of causal inference approaches for observational studies, we are able to include patients with standard care from the past, reducing the sample size and the number of patients exposed to the study setting. Also, since women receiving standard care are uninformed of new interventions, we believe the current approach more accurately represents normal practice, while putting as little burden on the women and caregiver as possible. To account for potential differences between the historical and prospective cohorts, including changes in clinical practice over time (eg, due to COVID-19), we applied a propensity score adjustment strategy and conducted sensitivity analyses to ensure methodological robustness and reduce bias.

## Supplementary material

10.1136/bmjopen-2025-102732 online supplemental file 1
