# Supplementary material for: Continuous non-invasive electrophysiological monitoring in high-risk pregnancies: study protocol of a cohort intervention random sampling study in a tertiary obstetrical care centre in the Netherlands (NIEM-O study)
Source: BMJ Open. 2025 Nov 12;15(11):e102732. doi: 10.1136/bmjopen-2025-102732 (PMC12612757; doi:10.1136/bmjopen-2025-102732)
Supplement: online supplemental file 1 [file bmjopen-15-11-s001.pdf]

Appendix F: Informed consent form – subject

Belonging to the NIEMO-study: A medical study on external monitoring of pregnant women during admission to the Obstetric High Care.

- I have read the information sheet. I was able to ask questions. My questions have been answered well enough. I had enough time to decide if I wanted to take part.
- I know that taking part is voluntary. I also know that at any time I can decide not to take part in the study. Or to stop taking part. I do not have to explain why.
- I give the investigator consent to inform and contact specialist (s) who treats me that I am taking part in this study and to retrieve my and my child’s data after birth, that are requisite for the research.
- I give consent to give my doctor or specialist information about accidental discoveries made during the study that are important for my health.
- I give consent to collect and use my data. The investigators only do this to answer the question of this study.
- I know that some people will be able to see all of my data to review the study. These people are mentioned in this information sheet. I give consent to let them see my data for this review.
- I give consent to collect my data, when no NFMS is available or I don’t want to receive the NFMS, and I receive standard treatment

- Please tick yes or no in the table below.

I give consent to share my coded data with other researchers	Yes☐	No☐
I give consent to store my data to use for other research, as stated in the information sheet.	Yes☐	No☐
I give consent to ask me after this study if I want to participate in a follow-up study.	Yes☐	No☐
I give consent to share some of my coded data with Nemo B.V. in order to improve quality of monitoring	Yes☐	No☐

- I want to take part in this study.

My name is (subject): ………………………………..
Signature: ……………………… Date : __/__/__

If there is another (authoritative) parent, legal representative(s) or guardian besides you (the mother), we also ask that person to give separate permission for the collection of your child's data after birth

Is there is another (authoritative) parent, legal representative(s) or guardian besides you?

☐ Yes
☐ No

If you answered YES, they must sign below for permission. If that person does not give permission, we are not allowed to collect your child's data.

The name of the other (authoritative) parent, legal representative(s) or guardian is:
……………………………………………………………………………………………………..

Signature: …………………………… Date: __ / __ / __

I declare that I have fully informed this subject about the study mentioned.

If any information becomes known during the study that could influence the subject's consent, I will let this subject know in good time.

Investigator name (or their representative): ....
Signature:……………………… Date: __/__/__

The study subject will receive a complete information sheet, together with a signed version of the consent form.
