# Supplementary material for: Continuous non-invasive electrophysiological monitoring in high-risk pregnancies: study protocol of a cohort intervention random sampling study in a tertiary obstetrical care centre in the Netherlands (NIEM-O study)
Source: BMJ Open. 2025 Nov 12;15(11):e102732. doi: 10.1136/bmjopen-2025-102732 (PMC12612757; doi:10.1136/bmjopen-2025-102732)
Supplement: Supplementary file 2 [file bmjopen-2025-102732.reviewer_comments.pdf]

PEER REVIEW HISTORY

BMJ Open publishes all reviews undertaken for accepted manuscripts. Reviewers are asked to complete a checklist review form and are provided with free text boxes to elaborate on their assessment. These free text comments are reproduced below.

ARTICLE DETAILS

Title (Provisional)

Continuous non-invasive electrophysiological monitoring in high-risk pregnancies: study protocol of a cohort intervention random sampling study in a tertiary obstetrical care centre in the Netherlands (NIEM-O study)

Authors

de Klerk, Nadine Denice; Berben, Phebe B Q; de Vries, I R; Niemarkt, Hendrik; Vullings, Rik; van den Heuvel, Edwin; van der Ven, M; Fransen, A F; Oei, Guid; van Laar, J. O.E.H.

VERSION 1 - REVIEW

**Reviewer**	**1**
**Name**	**Cavoretto, Paolo**
**Affiliation**	**Univ Vita Salute San Raffaele, Obstetrics and Gynaecology**
**Date**	**04-Aug-2025**
**COI**	**None**

The methods section of the NIEM-O study demonstrates careful planning and a clear intent to evaluate the potential benefits of continuous electrophysiological fetal monitoring (eCTG) in a high-risk obstetric population. The study's innovative use of the Cohort Intervention Random Sampling Study (CIRSS) design is commendable, it allows for real-world applicability, greater inclusion, and reduced patient burden, which are key strengths in perinatal research. Overall, the study design is thoughtful and well-articulated. Addressing these areas would enhance the rigour and clarity of this important work.

There are a few areas where additional clarity or adjustment could strengthen the protocol further:

1. Justification for CIRSS over RCT: While the CIRSS model has practical advantages, the decision to forego a randomized controlled trial may compromise internal validity. A clearer rationale addressing potential biases and how they will be controlled (beyond propensity scoring) would enhance the reader's confidence.

2. Handling of signal quality limitations: The potential for signal loss due to vernix caseosa (particularly between 28–32 weeks) is acknowledged, but the study lacks a predefined threshold or protocol for switching devices beyond the 20% signal loss rule from intrapartum care. More detail on how such decisions will be standardized would improve reproducibility.

3. Interobserver variability: Given that CTG interpretation is inherently subjective and especially challenging in preterm cases, it would be beneficial to describe any training, calibration, or consensus procedures for the clinical team. This would help address variability and ensure more consistent data interpretation.

4. Patient feedback timing: Surveys assessing patient satisfaction are planned at admission and discharge. Consider adding a short follow-up (e.g., post-discharge) to capture reflections that may emerge after hospital stay.

5. Use of historical controls: While pragmatic, the historical cohort may differ systematically from current patients (e.g., in clinical practice changes post-COVID). Further detail on how this will be adjusted or monitored over time would strengthen the methodological transparency.

6. Clarify methods and resources to monitor these patients 21 hours per day. This will involve an important effort.

Reviewer **2**

Name **Ueda, Kayo**

Affiliation **Nagasaki University Graduate School of Biomedical Sciences, Reproductive Health, Institute of Biomedical Sciences**

Date **05-Sep-2025**

COI **None**

Overall assessment

I would like to thank the editors for the opportunity to review this study protocol. It is a privilege to contribute to the evaluation of such an important and clinically relevant research project.

This study protocol describes the NIEM-O study, which evaluates the effect of continuous antepartum non-invasive electrophysiological monitoring (eCTG) compared with conventional intermittent CTG in high-risk preterm pregnancies. Conventional CTG has notable limitations in monitoring preterm fetuses, and continuous eCTG may represent an important innovation. The topic is clinically meaningful and timely. The study design is generally appropriate, with ethical approval and trial registration clearly documented.

Strengths

• The adoption of the CIRSS (Cohort Intervention Random Sampling Study) design is pragmatic and enables inclusion of a large and representative patient cohort.

• The use of historical cohorts reduces the number of newly recruited participants and limits the burden on both patients and providers.

• Patient and public involvement (Care4Neo) is commendable and ensures that patient-centered perspectives are incorporated.

• The statistical analysis plan appropriately includes causal inference approaches such as propensity score adjustment, as well as sensitivity and subgroup analyses.

• Ethical approval, trial registration, and detailed descriptions of the monitoring device are provided, ensuring transparency.

Limitations / Concerns

• As the CIRSS design is not fully randomized, there is an inherent risk of confounding. Baseline differences between the intervention and standard care groups are possible, making appropriate adjustment using causal inference methods (e.g., propensity scores) essential.

• The use of historical cohorts may introduce bias due to secular changes in clinical practice and treatment policies; clearer explanation of how these factors will be addressed would strengthen the protocol.

• It would be advisable to explicitly acknowledge that, while efforts are made to approximate the validity of an RCT, confounding cannot be entirely eliminated.

• The assumed effect size for the sample size calculation appears to be largely based on clinical experience; supporting evidence from the literature would improve justification.

• Signal quality issues between 28 and 32 weeks' gestation are noted, but the definition of insufficient signal and criteria for switching to conventional CTG should be made clearer.

• Patient- and caregiver-reported outcomes are well chosen, but further detail on handling of missing data and multiple testing for secondary endpoints would be beneficial.

Suggestions for improvement

• Provide a clearer description of the random sampling procedure (e.g., who conducts the sampling and at what stage).

• Specify the covariates to be included in propensity score models (maternal characteristics, fetal indicators, care-related variables, etc.).

• Clarify how secular changes between historical and prospective cohorts will be accounted for in the analysis.

• Expand on how thresholds for signal loss will be applied in practice and how this will be reported.

Conclusion

This study addresses an important clinical question and presents an innovative protocol with the potential to inform future practice. Overall, the design is sound, but additional clarification of certain methodological and analytical details would further enhance transparency and rigor. I therefore recommend minor revision.

VERSION 1 - AUTHOR RESPONSE

Reviewer 1 - Dr. Paolo Cavoretto, Univ Vita Salute San Raffaele

The methods section of the NIEM-O study demonstrates careful planning and a clear intent to evaluate the potential benefits of continuous electrophysiological fetal monitoring (eCTG) in a high-risk obstetric population. The study's innovative use of the Cohort Intervention Random Sampling Study (CIRSS) design is commendable, it allows for real-world applicability, greater inclusion, and reduced patient burden, which are key strengths in perinatal research. Overall, the study design is thoughtful and well-articulated. Addressing these areas would enhance the rigour and clarity of this important work.

There are a few areas where additional clarity or adjustment could strengthen the protocol further:

1. Justification for CIRSS over RCT: While the CIRSS model has practical advantages, the decision to forego a randomized controlled trial may compromise internal validity. A clearer rationale addressing potential biases and how they will be controlled (beyond propensity scoring) would enhance the reader's confidence.

We thank the reviewer for their insightful comment regarding the choice of study design. While randomized controlled trials (RCTs) are considered the gold standard for internal validity, they often face limitations in external validity, recruitment feasibility, and ethical concerns—particularly in vulnerable populations such as high-risk pregnant women. The CIRSS design was selected to address these limitations by combining the strengths of observational cohort studies and pragmatic trial elements. To further substantiate this choice, we refer to our recently published methodological paper in the Journal of Comparative Effectiveness Research (de Klerk et al., 2025), which provides a comprehensive rationale for the CIRSS design, including its ethical, statistical, and logistical advantages over traditional RCTs. In particular, CIRSS allows for real-world applicability, minimizes

patient burden, and enables the inclusion of historical controls, while still maintaining internal validity through causal inference techniques such as propensity score adjustment and per-protocol analysis.

Reference:
de Klerk ND, Berben PBQ, van der Ven M, et al. Optimizing clinical scientific research: the cohort intervention random sampling study with historical controls. J Comp Eff Res. 2025; e240168. doi: 10.57264/cer-2024-0168.

We have added the following in the manuscript to clarify this: "*The cohort intervention random sampling study (CIRSS) was chosen to balance methodological rigour with feasibility and ethical considerations in a high-risk obstetric population. This design features has recently been described and compared with other study designs[34], highlighting its ability to combine real-world applicability with robust statistical correction for potential biases. The use of historical controls and random sampling within a prospective cohort allows for efficient implementation and broader inclusion, while maintaining internal validity through causal inference techniques such as inverse probability weighting with a per-protocol analysis".* Line 249-258.

2. Handling of signal quality limitations: The potential for signal loss due to vernix caseosa (particularly between 28–32 weeks) is acknowledged, but the study lacks a predefined threshold or protocol for switching devices beyond the 20% signal loss rule from intrapartum care. More detail on how such decisions will be standardized would improve reproducibility.

We appreciate the reviewer's attention to signal quality. The 20% signal loss threshold, derived from FIGO intrapartum guidelines, will be used as a benchmark. To ensure reproducibility, we will implement a standardized protocol for switching to conventional CTG, based on clinical judgment supported by predefined criteria. In the manuscript, this now reads as:
"*Based on this, a standardized protocol for switching to conventional CTG will be implemented when signal loss exceeds 20%, or when clinical judgment deems the eCTG trace insufficient for reliable interpretation. This protocol will be documented and monitored throughout the study."* Line 306-311.

3. Interobserver variability: Given that CTG interpretation is inherently subjective and especially challenging in preterm cases, it would be beneficial to describe any training,

calibration, or consensus procedures for the clinical team. This would help address variability and ensure more consistent data interpretation.

We thank the reviewer for highlighting the importance of consistency in CTG interpretation, particularly in preterm cases. All clinical staff involved in CTG assessment are trained according to standard hospital procedures, which include systematic evaluation of CTG traces based on current literature and internal guidelines. See line 492-502: "*Before the study starts, medical staff will be trained and standard operating procedures (SOP) – including recommendations in interpretation of preterm CTG traces – will be made by the research team based on available literature [46,49–53]. Additionally, CTG traces will be discussed during daily meetings between the attending gynecologists."* In order to further clarify this, this text was altered as it now reads:

"Before study initiation, all clinical staff involved in CTG interpretation will undergo standardized training sessions based on current literature and internal guidelines. Additionally, daily multidisciplinary meetings will be held to discuss CTG traces, fostering consensus and calibration among attending gynecologists. These procedures will be documented in a Standard Operating Procedure (SOP). Line 492-502

4. Patient feedback timing: Surveys assessing patient satisfaction are planned at admission and discharge. Consider adding a short follow-up (e.g., post-discharge) to capture reflections that may emerge after hospital stay.

While the timing of the satisfaction surveys is fixed—at admission and discharge—we would like to clarify that patients are followed up according to protocol until four weeks postpartum.

"*Although the patient satisfaction surveys are administered at admission and discharge, all participants are followed up until (at least) four weeks postpartum. During this period, patients have the opportunity to share additional reflections or concerns with their care team, which may provide further qualitative insights into their experience with the monitoring method.*"

This clarification is added to the manuscript in line 318-323.

5. Use of historical controls: While pragmatic, the historical cohort may differ systematically from current patients (e.g., in clinical practice changes post-COVID). Further detail on how this will be adjusted or monitored over time would strengthen the methodological transparency.

We thank the reviewer for raising this important point. As correctly noted, the use of historical controls may introduce systematic differences due to temporal changes in clinical practice, including those following the COVID-19 pandemic.

"To address this, the study protocol includes a detailed statistical plan involving propensity score adjustment for both primary and secondary outcomes. This approach accounts for differences in baseline characteristics between the historical and prospective cohorts, as well as between women who were offered eCTG but received CTG. Furthermore, sensitivity analyses will be conducted to assess the robustness of findings across subgroups and time periods. These measures are intended to enhance methodological transparency and mitigate potential bias."

This is already described in the Statistical Analysis section (from line 402). In order to reinforce this, clearly emphasized in the Discussion (line 523-527), where it reads: "*To account for potential differences between the historical and prospective cohorts, including changes in clinical practice over time (for example due to COVID-19), we applied a propensity score adjustment strategy and conducted sensitivity analyses to ensure methodological robustness and reduce bias."*

6. Clarify methods and resources to monitor these patients 21 hours per day. This will involve an important effort.

We recognize the logistical complexity of continuous monitoring. To clarify our approach, we propose the following addition:

"For continuous monitoring, trained nursing staff will oversee device placement and signal quality checks during routine care. CTG traces are continuously displayed on a central monitoring system, where deviations from normal patterns trigger automated alarms to alert clinical staff. In addition, CTG traces are systematically assessed by physicians at least four times per 24 hours at predefined intervals, partly retrospectively, and more frequently if clinically indicated. These assessments follow a Standard Operating Procedure (SOP) to ensure consistency and clinical relevance." Line 291-299.

Reviewer 2 - Dr. Kayo Ueda, Nagasaki University Graduate School of Biomedical Sciences, Kyoto University School of Public Health

Strengths

• The adoption of the CIRSS (Cohort Intervention Random Sampling Study) design is pragmatic and enables inclusion of a large and representative patient cohort.

• The use of historical cohorts reduces the number of newly recruited participants and limits the burden on both patients and providers.

• Patient and public involvement (Care4Neo) is commendable and ensures that patient-centered perspectives are incorporated.

• The statistical analysis plan appropriately includes causal inference approaches such as propensity score adjustment, as well as sensitivity and subgroup analyses.

• Ethical approval, trial registration, and detailed descriptions of the monitoring device are provided, ensuring transparency.

Limitations / Concerns

• As the CIRSS design is not fully randomized, there is an inherent risk of confounding. Baseline differences between the intervention and standard care groups are possible, making appropriate adjustment using causal inference methods (e.g., propensity scores) essential.

• The use of historical cohorts may introduce bias due to secular changes in clinical practice and treatment policies; clearer explanation of how these factors will be addressed would strengthen the protocol.

• It would be advisable to explicitly acknowledge that, while efforts are made to approximate the validity of an RCT, confounding cannot be entirely eliminated.

• The assumed effect size for the sample size calculation appears to be largely based on clinical experience; supporting evidence from the literature would improve justification.

• Signal quality issues between 28 and 32 weeks' gestation are noted, but the definition of insufficient signal and criteria for switching to conventional CTG should be made clearer.

• Patient- and caregiver-reported outcomes are well chosen, but further detail on handling of missing data and multiple testing for secondary endpoints would be beneficial.

Suggestions for improvement

1. Provide a clearer description of the random sampling procedure (e.g., who conducts the sampling and at what stage).

We thank the reviewer for the insightful remarks, and would like to refer to the following information regarding the random sampling procedure in the manuscript (line 265-287):

Potential participants will be informed about the study by their obstetric healthcare professional when admitted to the hospital. Firstly, the health care professionals will verify the in- and exclusion criteria (Table 1), after which the women are added to the study using an encryption key. These women are part of the cohort study and receive standard care existing of conventional intermittent CTG monitoring. A part of these women will be randomly sampled. This random sampling is conducted by the research team using a secure algorithm embedded in Research Manager (ISO 27001 certified). They will be offered the new treatment: continuous eCTG monitoring using the NFMS (Figure 2). The women will either be part of the cohort study, thus receiving standard care existing of conventional intermittent CTG monitoring, or they will be sampled for continuous eCTG monitoring using the NFMS. Appropriate explanation and written study information will be provided hereafter by the health care professional, and they will ask the researchers for further counselling. Non-sampled women will only receive information regarding the collection of their data in the cohort study. Sampled women will be informed about receiving continuous eCTG monitoring. A researcher will complete the Informed Consent (IC) procedure, including collecting written consent (for an example of the participant consent form, see Supplementary File 1). When the women do not wish to participate in the study, they will be excluded, their encrypted information will be obliterated and the exclusion will be anonymously documented.

In order to further clarify this, the following sentences was added:

"*This random sampling is conducted by the research team using a secure algorithm embedded in Research Manager (ISO 27001 certified).*" Line 271-272.

Additionally, per previous remark (comment 1) of the other reviewer, we referred to a recently published article [de Klerk et al. 2025] regarding the CIRSS design, in which IC procedures are also described more extensively, for further clarification.

2. Specify the covariates to be included in propensity score models (maternal characteristics, fetal indicators, care-related variables, etc.).

We agree that specifying the covariates used in the propensity score models enhances methodological clarity. Therefore, we have added the following to the manuscript:

"The propensity score models will include maternal characteristics (e.g., age, BMI, parity, ethnicity), foetal indicators (e.g., gestational age, foetal growth restriction, Doppler abnormalities), and care-related variables (e.g., indication for admission, medication use, duration of hospitalization). These covariates were selected based on clinical relevance and availability across both cohorts." Line 409-414.

3. Clarify how secular changes between historical and prospective cohorts will be accounted for in the analysis.

We appreciate the reviewer's concern regarding potential bias introduced by secular changes in clinical practice. A similar point was raised by Reviewer 1 (Comment 5), to which we responded by detailing our approach using propensity score adjustment and sensitivity analyses.

"To address potential secular changes in clinical practice, including those related to evolving treatment protocols and the impact of the COVID-19 pandemic, time-period indicators will be incorporated into the propensity score models. Additionally, stratified sensitivity analyses will be conducted to assess the robustness of findings across different time frames."

This approach is already described in the Statistical Analysis section (from line 402). To reinforce this, it is also clearly emphasized in the Discussion (lines 523-527), where it reads:

"To account for potential differences between the historical and prospective cohorts, including changes in clinical practice over time (for example due to COVID-19), we applied a propensity score adjustment strategy and conducted sensitivity analyses to ensure methodological robustness and reduce bias."

We trust this adequately addresses the concern and will ensure these points remain clearly visible in the final version of the manuscript.

4. Expand on how thresholds for signal loss will be applied in practice and how this will be reported.

We appreciate the reviewer's request for further clarification regarding signal quality thresholds and

switching protocols. A similar concern was raised by Reviewer 1 (Comment 2), to which we responded:

“The 20% signal loss threshold, derived from FIGO intrapartum guidelines, will be used as a benchmark. To ensure reproducibility, we will implement a standardized protocol for switching to conventional CTG, based on clinical judgment supported by predefined criteria.

In the manuscript, this now reads as: *“Based on this, a standardized protocol for switching to conventional CTG will be implemented when signal loss exceeds 20%, or when clinical judgment deems the eCTG trace insufficient for reliable interpretation. This protocol will be documented and monitored throughout the study.”* Line 306-311.”

We trust this clarification ensures reproducibility and addresses the reviewer’s concern.
